# Relationship between gestational body mass index change and the risk of gestational diabetes mellitus: a community-based retrospective study of 41,845 pregnant women

**DOI:** 10.1186/s12884-022-04672-5

**Published:** 2022-04-19

**Authors:** Shuang Zhang, Huikun Liu, Nan Li, Wei Dong, Weiqin Li, Leishen Wang, Yu Zhang, Yingzi Yang, Junhong Leng

**Affiliations:** 1 Tianjin Women’s and Children’s Health Center, No. 96 Guizhou Road, Heping District, Tianjin, 300070 China; 2 Department of Epidemiology and Biostatistics, School of Public Health, Tianjin Medical University, Tianjin, 300070 China

**Keywords:** Gestational diabetes mellitus, Body mass index, Community-based study

## Abstract

**Background:**

Gestational diabetes mellitus (GDM) is associated with adverse health consequences for women and their offspring. It is associated with maternal body mass index (BMI) and may be associated with gestational weight gain (GWG). But due to the heterogeneity of diagnosis and treatment and the potential effect of GDM treatment on GWG, the association between the two has not been thoroughly clarified. Compared to body weight, BMI has the advantage that it considers height during the whole course of pregnancy. Understanding BMI changes during pregnancy may provide new evidence for the prevention of GDM.

**Methods:**

This study investigated the BMI change of pregnant women based on a retrospective study covering all communities in Tianjin, China. According to the results of GDM screening at 24–28 weeks of gestation, pregnancies were divided into the GDM group and the non-GDM group. We compared gestational BMI change and GWG in the two groups from early pregnancy to GDM screening. GWG was evaluated according to the IOM guidelines. Logistic regression was applied to determine the significance of variables with GDM.

**Results:**

A total of 41,845 pregnant women were included in the final analysis (GDM group, *n* = 4257 vs. non-GDM group, *n* = 37,588). BMI gain has no significant differences between the GDM and non-GDM groups at any early pregnancy BMI categories (each of 2 kg/m^2^), as well as weight gain (*P* > 0.05). Early pregnancy BMI was a risk factor for GDM (*OR* 1.131, *95% CI* 1.122–1.139). And BMI gain was associated with a decreased risk of GDM in unadjusted univariate analysis (*OR* 0.895, *95% CI* 0.869–0.922). After adjusting on early pregnancy BMI and other confounding factors, the effect of BMI gain was no longer significant (*AOR* 1.029, *95% CI* 0.999–1.061), as well as weight gain (*AOR* 1.006, *95% CI* 0.995–1.018) and GWG categories (insufficient: *AOR* 1.016, *95% CI* 0.911–1.133; excessive: *AOR* 1.044, *95% CI* 0.957–1.138).

**Conclusions:**

BMI in early pregnancy was a risk factor for GDM, while BMI gain before GDM screening was not associated with the risk of GDM. Therefore, the optimal BMI in early pregnancy is the key to preventing GDM.

**Supplementary Information:**

The online version contains supplementary material available at 10.1186/s12884-022-04672-5.

## Background

Gestational diabetes mellitus (GDM) is a common metabolic complication of pregnancy defined as glucose intolerance first identified during pregnancy. It increases the risk of adverse pregnancy outcomes, such as preterm birth, cesarean delivery, macrosomia, postpartum type 2 diabetes mellitus, and metabolic diseases in offspring [1–6]. The prevalence of GDM is increasing rapidly worldwide along with the lifestyle changes, growing incidence of obesity, and older age of pregnant women [7, 8]. It currently affects 3–25% of pregnancies worldwide, constituting a significant global healthcare burden [9]. A meta-analysis review suggests that the total incidence of GDM in China is 14.8% [10]. It increased almost 3.5-fold from 1999 to 2012 according to the data of universal screening for GDM in Tianjin, China [11–13]. Genetic and environmental factors jointly promote its onset [14, 15]. Previous studies have helped to identify a multitude of potential risk factors for GDM. These include advancing maternal age, increasing pre-pregnancy body mass index (BMI), increasing parity, having a previous macrosomia baby, family history of diabetes, polycystic ovarian syndrome (PCOS), and habitual smoking [7, 13, 16, 17]. More attention should be paid to the prevention and control of GDM.

Lifestyle changes are essential in the management of GDM. The cornerstone of GDM treatment is medical nutrition therapy (MNT), together with weight management and physical exercise [18]. These measures have beneficial effects on glucose and insulin levels and can contribute to better pregnancy outcomes [19, 20]. Some studies have found that diet and exercise interventions during pregnancy could reduce risks of GDM [7, 21], and this effect may be relevant to the lifestyle improvements at the beginning of pregnancy that decrease the gestational weight gain (GWG) before the mid-second trimester [22–24]. However, today there are still many disputes, even regarding current indications.

During gestation, women experience a series of physical and metabolic modifications and adaptations, which aim to protect the fetus’s development and are closely related to both prepregnancy nutritional status and GWG [18]. The negative effects of both insufficient and excessive GWG on maternal-fetal outcomes have been taken into account by the IOM that developed universal guidelines for optimal GWG based on prepregnancy BMI categories [25, 26]. GWG below guidelines in the United States, Europe, and Asia was 21, 18, and 31%, and above was 51, 51, and 37% respectively [27]. The risks associated with excess GWG may be higher in women from Asia [27]. Regional BMI categories are acknowledged to be more applicable than WHO BMI categories when applying IOM GWG guidelines in the Asia population [27].

GWG is a modifiable risk factor for adverse pregnancy outcomes. Weight assessment in the first and second trimesters contributes to early identification, prevention, and intervention for adverse perinatal outcomes. GDM is related to maternal BMI and possibly to GWG, associations could not be assessed because of heterogeneity of diagnosis and treatment as well as the potential effect of GDM treatment on GWG [25, 28–30]. Many studies support that overweight and obesity before pregnancy and an excessive GWG are associated with a greater risk of developing GDM [31–34]. Recently, Chinese researchers report that women with excessive GWG had a significantly 32.8% increased risk of developing GDM compared with non-excessive GWG [35]. But some studies from the United States found that women with and without GDM had similar mean GWG before GDM screening [36, 37]. Furthermore, other studies from China have found an association of GWG above guidelines with a lower risk of GDM [38, 39]. Therefore, the association between GWG and the risk of GDM needs further confirmation.

Compared to body weight, BMI has the advantage that it considers height during the whole course of pregnancy. Prepregnancy BMI has been proved the main predictor of GDM. But the relationship between the change of BMI during pregnancy and the risk of GDM has not been elucidated. Previous studies have found that inter-pregnancy BMI change may be associated with the risk of obstetric complications [40, 41]. So we focus on the BMI before GDM screening and are committed to providing a new measurement to evaluate the relationship between energy balance during pregnancy and health outcomes. This just reflects the new insight and practical value of this study.

We aimed to investigate the relationship between inter-gestational BMI on the risk of GDM, and a better understanding of it is vital for developing evidence-based interventions and guidance.

## Methods

### Population and data collection

This study was based on a public women and children’s health care system in Tianjin, China. In Tianjin, more than 80,000 pregnant women attend antenatal care each year, and antenatal care coverage is maintained at over 95%. In this study, all the prenatal medical information was retrospectively collected from the Tianjin Women and Children Health Information System (TJWCHIS) database, and the data was anonymized. The study protocol was approved by the Human Subjects Committee of the Tianjin Women’s and Children’s Health Center. All methods were carried out in accordance with relevant guidelines and regulations. Since this was a retrospective analysis of data routinely collected from participants, the consent for participation was not applicable. The need for informed consent was waived by the Human Subjects Committee of the Tianjin Women’s and Children’s Health Center.

Basic characteristics of pregnant women were collected at the first antenatal visit. It included maternal age, ethnicity, education, gravidity, parity, history of diabetes, hypertension, PCOS, obstetrical history (e.g., history of macrosomia, infant death), family history of diabetes or hypertension, and lifestyle habits (e.g., habitual smoking). We included women aged 18–45 years with singletons pregnancy. All of them were followed up to measure their weight from early pregnancy to GDM screening. To avoid the influence of pre-pregnancy diseases on the results, we excluded women with diabetes or hypertension before pregnancy. In this study, all pregnant women were tested for blood glucose during the first antenatal examination. If the results met the criteria for diagnosis of diabetes in pregnancy (DIP), they were not included in the analysis. The criteria were: FG ≥7.0 mmol/L, and/or 2-h 75 g oral glucose tolerance test (OGTT) value ≥11.1 mmol/L, or random plasma glucose ≥11.1 mmol/L associated with signs and symptoms of diabetes [42] (Additional Fig. 1).

### Screening and diagnosis of GDM

At present, the screening strategy and diagnostic criteria of GDM were inconsistent in various countries and regions [43]. In this study, a two-step strategy was used to screen for GDM at the 24th–28th week of gestation [13, 44]. The first step: perform a 50 g glucose challenge test (GCT) to measure the plasma glucose level at 1 h after the glucose load. If the plasma glucose level was ≥7.8 mmol/L, the pregnant woman will be required to perform a 75 g OGTT. The second step: The 75 g OGTT should be performed when the pregnant women were fasting for 10–12 h. At 8–9 am peripheral blood glucose levels of fasting, 1 and 2 h after taking glucose were measured. If at least one of them exceeded the thresholds of 5.1, 10.0, and 8.5 mmol/L at fasting, 1, and 2 h respectively, GDM was diagnosed [45]. Based on the results, pregnant women were classified as the GDM group and the non-GDM group.

### Anthropometric measurement

Weight was measured at the first prenatal visit (mean for gestational weeks when the measurement was conducted) and the time of GDM screening (mean for gestational weeks when the measurement was conducted). While bodyweight at the first prenatal visit was used to calculate early pregnancy BMI, bodyweight at the time of GDM screening was used to calculate the BMI at the late second trimester. BMI was calculated by dividing weight by height squared. The BMI gain was calculated by the formula of BMI gain = BMI at the late second trimester - early pregnancy BMI.

The rate of weight gain at the second trimester was calculated as follows: (weight measured at the time of GDM screening − weight measured at the first prenatal visit – first-trimester weight gain) / (gestational age of weight measured at the time of GDM screening – 13 weeks). Weight gain throughout pregnancy followed a non-linear trajectory. The rate of weight gain was greater in the second than in the first half of pregnancy [46]. The average weight gain during the first trimester was assumed to be 0.5-2 kg [26]. Therefore, we divided pregnant women into three categories (insufficient, adequate, or excessive weight gain) based on their rate of weight gain at the second trimesters. According to the Institute of Medicine (IOM) guidelines, the recommended rate of weight gain in the second and third trimesters was 0.44–0.58, 0.35–0.50, 0.23–0.33, and 0.17–0.27 kg/week in the underweight, normal weight, overweight, and obese groups, respectively [26]. And BMI categories were commented the Chinese BMI criteria: underweight (BMI < 18.5 kg/m^2^), normal weight (BMI 18.5–23.9 kg/m^2^), overweight (BMI 24.0–28.0 kg/m^2^), and obese (BMI ≥ 28.0 kg/m^2^), respectively. Either self-reported prepregnancy or measured weight in the first trimester was usually used for calculating prepregnancy BMI and GWG [26]. But the accuracy of self-reported prepregnancy weight has been questioned, so we performed BMI categories based on the BMI calculated by measured weight at early pregnancy.

### Statistical analysis

The analyses were performed using IBM SPSS Statistics (Version 21.0). The figures were drawn using GraphPad Prism (Version 8.0). Normal continuous variables were expressed by means (SD), which were compared between two or more groups using a *t*-test of independent samples or one-way analysis of variance (ANOVA). Categorical variables were described as numbers (percentage) and compared by the *Chi-square* test. Binary logistic regression analysis was used to demonstrate the effect of the factors on GDM. BMI gain and weight gain were adjusted for the gestational week of weight measurement. The potential risk factors of GDM included FG in the first trimester, blood pressure, age, multipara, PCOS, history of macrosomia, history of adverse fertility, family history of diabetes, and habitual smoking. The factors confirmed by univariate analysis will be adjusted as confounding factors in multiple analyses. A two-sided *P*-value of less than 0.05 was considered a statistically significant difference. Multiple imputations were performed for missing values.

## Results

### Characteristics of pregnant women

There were 41,845 pregnant women (GDM group, *n* = 4257 vs. non-GDM group, *n* = 37,588) eligible for inclusion in the final analysis, with a mean (SD) age of 27.62 (4.10) years at study enrollment in 2015 (Additional Fig. 1). Women in the GDM group were older, had higher stature, body weight, BMI, FG in the first trimester, blood pressure (SBP, DBP, and mean arterial pressure (MAP)), and a higher proportion of multipara, PCOS, history of macrosomia, history of adverse fertility, family history of diabetes, and habitual smoking compared with the non-GDM group (*P* <  0.05) (Table 1). But women in the GDM group had significantly less BMI gain and rate of weight gain at the second trimester than those in the non-GDM group (*P* <  0.001).

**Table 1:** Characteristics of the Study Population

Characteristics	non-GDM group	GDM group	***P***-value
n (%)	37,588 (89.8)	4257 (10.2)	
Age, year	27.44 (4.05)	29.26 (4.15)	< 0.001
Height, cm	162.76 (4.79)	162.40 (4.98)	< 0.001
Weight at the first trimester, kg	59.64 (10.39)	64.65 (11.92)	< 0.001
BMI at the first trimester, kg/m^2^	22.49 (3.64)	24.49 (4.21)	< 0.001
Weight at the second trimester, kg	66.52 (10.36)	71.16 (11.67)	< 0.001
BMI at the second trimester, kg/m^2^	22.49 (3.64)	24.49 (4.21)	< 0.001
BMI gain, kg/m^2^	2.60 (1.07)	2.47 (1.14)	< 0.001
Rate of weight gain at the second trimester, kg/week	0.53 (0.24)	0.50 (0.25)	< 0.001
FG at the first trimester, mmol/L	4.76 (0.49)	4.96 (0.56)	< 0.001
SBP, mmHg	106.28 (10.31)	109.02 (10.84)	< 0.001
DBP, mmHg	68.97 (7.54)	70.89 (7.98)	< 0.001
MAP, mmHg	81.40 (7.95)	83.60 (8.43)	< 0.001
Multipara	10,888 (29.0%)	1324 (31.1%)	0.004
PCOS	13 (0.0%)	9 (0.2%)	< 0.001
History of macrosomia	41 (0.1%)	13 (0.3%)	0.001
History of adverse fertility	4536 (12.1%)	697 (16.4%)	< 0.001
Family history of diabetes	814 (2.2%)	193 (4.5%)	< 0.001
Habitual smoking (one or more cigarettes/day)	140 (0.4%)	29 (0.7%)	0.001

Data are means (SD) or n (%). MAP = (SBP + 2 × DBP)/3

*Abbreviation*s: *GDM* Gestational diabetes mellitus, *BMI* Body mass index, *FG* Fasting glucose, *SBP* Systolic blood pressure, *DBP* Diastolic blood pressure, *MAP* Mean arterial pressure, *PCOS* Polycystic ovarian syndrome

### Early pregnancy BMI and BMI gain

**Fig. 1:**
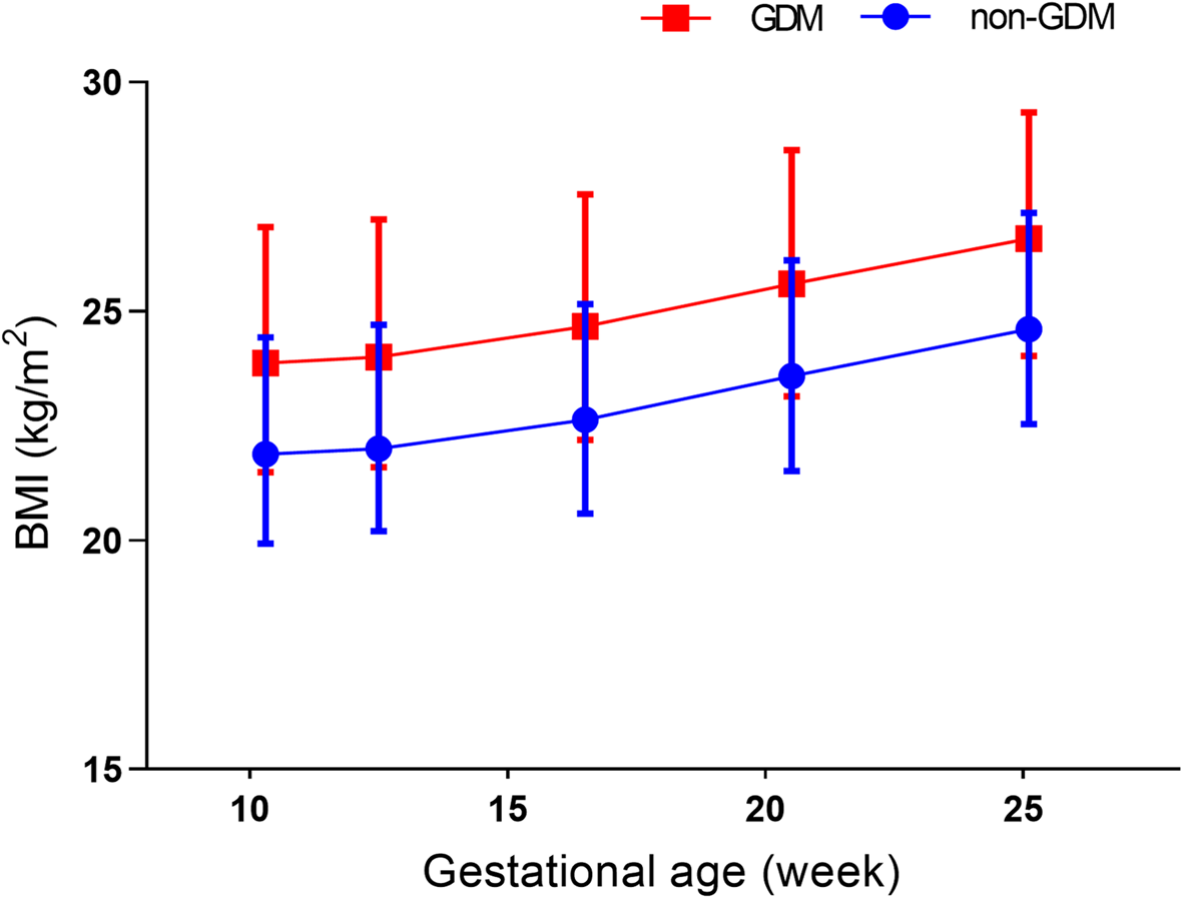
Comparison of BMI of pregnant women in the GDM group and the non-GDM group. * indicates *P* <  0.05, ** indicates *P* <  0.01; ns indicates *P* > 0.05. Data points were the means of maternal BMI, with the error bars corresponding to the standard deviation. The means of gestational age of weight measured were 10.3, 12.5, 16.4, 20.4, and 25.1 weeks, respectively. Abbreviation: GDM, gestational diabetes mellitus; BMI, body mass index

The early pregnancy BMI of women in the GDM group was significantly higher than that in the non-GDM group (*P* <  0.001) (Table 1). And this difference remained until GDM screening (Fig. 1). Table 2 showed that BMI gain decreased gradually with increasing early pregnancy BMI categories (each of 2 kg/m^2^) in the GDM group and the non-GDM group (*F* = 46.623, *P* <  0.001; *F* = 236.640, *P* <  0.001) (Additional Fig. 2). There was no significant difference between the two groups at any BMI categories (each of 2 kg/m^2^) (each *P* > 0.05) (Table 2).

**Table 2:** Comparison of BMI gain between the GDM and the non-GDM groups

Early pregnancy BMI (kg/m^2^)	*n*	BMI gain (kg/m^2^)		*t*	*P-value*
		non-GDM group	GDM group		
< 18.50	4080	2.74 (0.91)	2.70 (1.00)	0.592	0.554
18.50–19.99	6203	2.73 (0.95)	2.71 (1.03)	0.370	0.712
20.00–21.99	10,253	2.70 (1.00)	2.69 (1.00)	0.191	0.848
22.00–23.99	8507	2.68 (1.07)	2.65 (1.08)	0.755	0.450
24.00–25.99	5653	2.56 (1.10)	2.58 (1.13)	−0.391	0.696
26.00–27.99	3281	2.33 (1.19)	2.31 (1.11)	0.268	0.789
28.00–29.99	1845	2.15 (1.22)	2.10 (1.08)	0.760	0.447
≥30.00	2023	1.81 (1.29)	1.75 (1.29)	0.819	0.413
*F*		236.640	46.623		
*P-value*		< 0.001	< 0.001		

*Abbreviations*: *GDM* Gestational diabetes mellitus, *BMI* Body mass index

### Inadequate or excessive weight gain and GDM

**Fig. 2:**
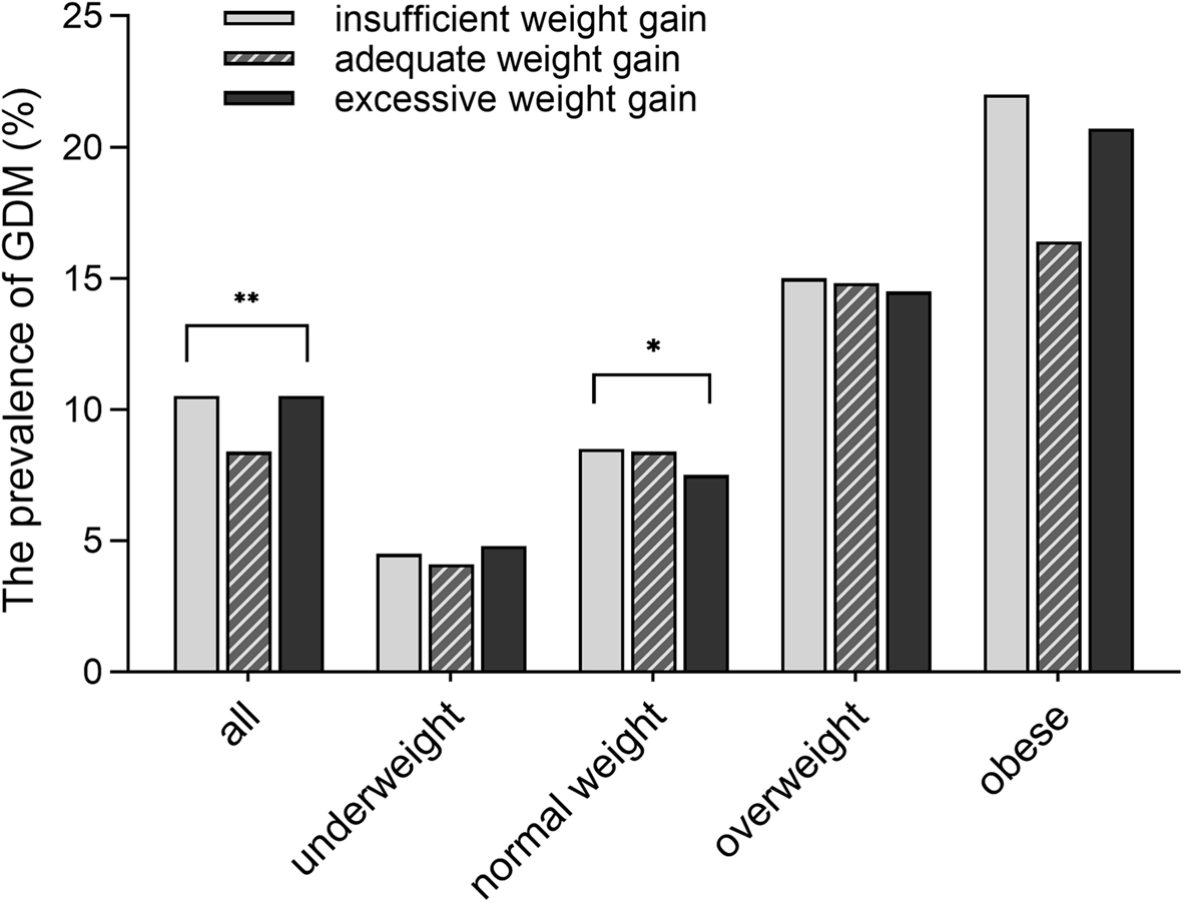
Comparison of the prevalence of GDM among different weight gain categories. Weight gain was evaluated according to the IOM guidelines based on the Chinese BMI categories. It recommended the optimal rate of weight gain at the second trimester was 0.44–0.58, 0.35–0.50, 0.23–0.33, and 0.17–0.27 kg/week in the underweight, normal weight, overweight, and obese groups, respectively. *Abbreviation: GDM, gestational diabetes mellitus; BMI, body mass index

The IOM guidelines are an adaptation of the most widely used criteria for the evaluation of GWG. According to the IOM’s recommendations, we divided 41,845 pregnant women into three categories: inadequate (*n* = 3340, 9.8%), appropriate (*n* = 11,227, 33.0%), or excessive (*n* = 19,406, 57.1%) based on their rate of weight gain at the second trimester. In general, women who gained insufficient or excessive weight had a significantly higher prevalence of GDM than women who gained adequate weight (10.5, 10.5%; vs 8.9%, *Chi-square* test, *P-value* <  0.001) (Fig. 2). But the BMI subgroup analysis showed that such differences were only significant in normal-weight women (8.5, 7.5%; vs 8.4%, *Chi-squared* test *P-*value = 0.026). And normal-weight women who gain too much weight seemed to have the lowest prevalence of GDM (Additional Table 1).

It led us to further analyze the effect of early pregnancy BMI on the results. Figure 2 showed that the prevalence of GDM was 4.5, 7.9, 14.6, and 20.5% in the underweight, normal weight, overweight, and obese group, respectively (*Chi-square* value = 927.931, *P-value* <  0.001). Early pregnancy BMI of pregnant women in each Chinese BMI category were compared between the GDM group and the non-GDM group. In the normal weight, overweight and obese group, the mean early pregnancy BMI of women with GDM was still significantly higher than that of women without GDM (*P* <  0.001) (Additional Table 2). And it could significantly affect the prevalence of GDM.

### Influencing factors of GDM

We performed logistic regression analysis to confirm the role of inter-gestational BMI on the risk of GDM (Table 3). The result showed that early pregnancy BMI was a risk factor for GDM (*OR* 1.131, *95% CI* 1.122–1.139). BMI gain was associated with a decreased risk of GDM in unadjusted univariate analysis (*OR* 0.895, *95% CI* 0.869–0.922), as well as the rate of weight gain (*OR* 0.956, 9*5% CI* 0.946–0.967). Both insufficient and excessive GWG contributed to a higher risk of GDM (*OR* 1.213, *95% CI* 1.093–1.347; *OR* 1.211, *95% CI* 1.115–1.316). It also showed that FG in the first trimester, MAP, age, multipara, PCOS, history of macrosomia, history of adverse fertility, family history of diabetes, and habitual smoking was all the risk factors of GDM (*P* <  0.05).

**Table 3:** Odds ratios (95% confidence intervals) of GDM by the effect of BMI gain and weight gain during pregnancy

Model	Factors	*β*	*OR*	*OR 95% C.I.*	*P*-value
Univariate analysis					
	Early pregnancy BMI	0.123	1.131	1.122–1.139	< 0.001
	BMI gain	−0.111	0.895	0.869–0.922	< 0.001
	Rate of weight gain	−0.045	0.956	0.946–0.967	< 0.001
	GWG evaluated by the IOM guideline				
	adequate				
	insufficient	0.193	1.213	1.093–1.347	< 0.001
	excessive	0.191	1.211	1.115–1.316	< 0.001
	FG in the first trimester	0.793	2.210	2.079–2.350	< 0.001
	MAP	0.034	1.034	1.030–1.038	< 0.001
	Age	0.105	1.111	1.102–1.119	< 0.001
	Multipara	0.102	1.107	1.034–1.186	0.004
	PCOS	1.812	6.124	2.616–14.334	< 0.001
	History of macrosomia	1.031	2.805	1.502–5.239	0.001
	History of adverse fertility	0.355	1.427	1.308–1.556	< 0.001
	Family history of diabetes	0.763	2.145	1.828–2.518	< 0.001
	Habitual smoking	0.607	1.835	1.228–2.740	0.003
Multivariate analysis					
Model 1	BMI gain	< 0.001	1.000	0.971–1.029	0.978
	Rate of weight gain	−0.004	0.996	0.985–1.007	0.472
	GWG evaluated by the IOM guideline				
	adequate				
	insufficient	0.007	1.007	0.905–1.121	0.897
	excessive	< 0.001	1.000	0.918–1.088	0.992
Model 2	BMI gain	0.030	1.030	1.000–1.061	0.050
	Rate of weight gain	0.006	1.006	0.995–1.017	0.273
	GWG evaluated by the IOM guideline				
	adequate				
	insufficient	−0.037	0.964	0.866–1.074	0.506
	excessive	0.027	1.027	0.943–1.119	0.537
Model 3	BMI gain	0.029	1.029	0.999–1.061	0.062
	Rate of weight gain	0.006	1.006	0.995–1.018	0.290
	GWG evaluated by the IOM guideline				
	adequate				
	insufficient	0.016	1.016	0.911–1.133	0.776
	excessive	0.043	1.044	0.957–1.138	0.333

Weight gain was evaluated according to the IOM guidelines based on the Chinese BMI categories. It recommended the optimal rate of weight gain at the second trimester was 0.44–0.58, 0.35–0.50, 0.23–0.33, and 0.17–0.27 kg/week in the underweight, normal weight, overweight, and obese groups, respectively

Model 1: adjusted for early pregnancy BMI

Model 2: adjusted for early pregnancy BMI, FG in the first trimester, and MAP

Model 3: adjusted for early pregnancy BMI, FG in the first trimester, MAP, age, multipara, PCOS, history of macrosomia, history of adverse fertility, family history of diabetes, and habitual smoking

*Abbreviation*s: *OR* Odds ratio, *CI* Confidence interval, *GDM* Gestational diabetes mellitus, *BMI* Body mass index, *GWG* gestational weight gain, *FG* Fasting glucose, *MAP* Mean arterial pressure, *PCOS* Polycystic ovarian syndrome

Then, a multiple regression equation was used to identify the actual influence of the factors with a stepwise selection of variables (Table 3). After adjusting for early pregnancy BMI, the effect of BMI gain was no longer significant (*AOR* 1.000, *95% CI* 0.971–1.029). The results of the analysis on the rate of weight gain at the second trimester (*AOR* 0.996, *95% CI* 0.985–1.007) and GWG categories (insufficient: *AOR* 1.007, *95% CI* 0.905–1.121; excessive: *AOR* 1.000, *95% CI* 0.918–1.088) were consistent with BMI gain. After additional adjustment for FG in the first trimester, MAP, and other confounding factors, the effect of BMI gain was still not significant (*AOR* 1.029, *95% CI* 0.999–1.061), as well as the rate of weight gain (*AOR* 1.006, *95% CI* 0.995–1.018) and GWG categories (insufficient: *AOR* 1.016, *95% CI* 0.911–1.133; excessive: *AOR* 1.044, *95% CI* 0.957–1.138).

### Sensitivity analyses

When exploring the effect of BMI gain on the risk of GDM, we also analyzed weight gain (Additional Table 3). The conclusions were consistent with BMI gain. And when evaluating GWG according to IOM guidelines, the WHO BMI categories were also conducted. The conclusions were consistent with the Chinese BMI categories (Additional Table 4).

## Discussion

To improve the health of mothers and their offspring, WHO has prioritized the achievement of ideal BMI before conception and prevention of excessive GWG. However, pre-gestational obesity represents a challenge of treatment, and nowadays there is new evidence as regards its management, especially the adequate GWG. Lifestyle interventions in pregnancy could help women attain recommended GWG. Optimal interventions and effects on outcomes are currently requiring research implementation [47]. Prior systematic reviews have not demonstrated that a healthy lifestyle and GWG reduced rates of GDM, even in high-risk populations [25]. It prompts us to rethink the implications of reducing GWG for the prevention of GDM.

This study investigated the correlation between maternal BMI and GDM by a large community-based cohort. The prevalence of GDM increases gradually with early pregnancy BMI (Fig. 2). And univariate logistic regression analysis also confirmed that BMI in early pregnancy was a risk factor for GDM (Table 3). Our results support the previous view that overweight and obesity significantly increase the risk of GDM [48, 49]. Women who develop GDM often have a subclinical metabolic dysfunction before pregnancy compared with women without GDM. Because of the significant decrease in insulin sensitivity in normal pregnancy, this predisposing initial insulin resistance is further exacerbated and, in combination with *β*-cell dysfunction, results in the development of GDM. However, today there are still many disputes, even regarding current indications.

This study found that BMI gain in the first months of pregnancy before GDM screening was not associated with GDM risk. Figure 1 showed that BMI increased in parallel between the GDM and non-GDM groups from the first to the second trimester. Independent sample *t*-test confirmed that there was no difference in BMI gain between the two groups. Furthermore, we also analyzed weight gain and got consistent results. This conclusion is consistent with some previous studies [36, 37], but it contradicts other studies [35, 38, 39]. This may need to be explained in terms of energy metabolism during pregnancy. GWG is the major determinant of the increase in energy demands during pregnancy. Although a total additional energy demand of about 76,000 kcal has been estimated during the whole pregnancy, there is an inter-individual variability of energy expenditure, linked not only to GWG but also to the pre-nutritional state, clear expression of the plasticity of the metabolic adaptations to the actual requirements [18]. But at present, the contribution of GWG to insulin resistance has not been clarified. The product of conception (placenta, fetus, amniotic fluid) accounts for about 35% of GWG [50]. Maternal body composition changes over the trimesters to support fetal growth. Maternal fat mass is the most variable component of GWG, which mostly contributes to the energy costs of pregnancy and positively correlates with GWG [51]. A previous study demonstrated that in obese women excessive GWG was associated with maternal fat, but not lean body mass accrual [52]. The results might explain why excess GWG is associated with long-term obesity and metabolic dysfunction. However, a variable change in the fat mass of the mother was mainly observed in the later stages of pregnancy [26, 50, 53]. In the first months of gestation, the changes in maternal body composition reflect the preparation of the maternal body for fetal development. Specifically, blood volume expands and the uterus and breast tissue of the maternal unit grows [18]. But mostly the accumulation of fat could be a reason for altered glucose tolerance. This may explain why excessive GWG in the first and second trimesters does not contribute to GDM by fat mass accrual. Therefore, the first half of BMI gain and weight gain was not associated with the risk of GDM.

Figure 2 showed that the rate of GWG greater than or less than the IOM guideline recommendations, compared with that within recommended levels, was associated with a higher risk of GDM. It meets the expectation of the IOM guidelines for reducing adverse pregnancy outcomes. But the BMI subgroup analysis showed that such differences were not significant in underweight, overweight, or obese women, respectively. This result suggested that GWG is not associated with the risk of GDM, and was consistent with our previous conclusion. Among women with normal weight in early pregnancy, those who gained too much weight gain had a lower risk of GDM than those who gained optimal weight. Similar observations have been reported in previous studies from Chinese populations [38, 39]. And Table 1 showed that BMI gain and GWG were both lower in the GDM group than in the non-GDM group. We further analyzed the causes of these results in detail. Next, we divided pregnant women based on BMI categories and found that the early pregnancy BMI of women with GDM was still significantly higher than the women without GDM in the normal weight, overweight and obese group, respectively (Additional Table 2). In addition, both BMI and weight gain decreased with initial BMI (Table 2, Additional Table 3). It could significantly affect the prevalence of GDM in different GWG evaluation categories. And this may explain why excess GWG has been associated with a lower risk of GDM in some previous studies [38, 39]. This view was also confirmed in multivariate logistic regression analysis. Neither insufficient nor excessive GWG affected the risk of GDM after adjusting for BMI in the first trimester. In addition, this result also suggested that we must be alert to the influence of initial BMI on the conclusion in future research on GWG. Since changes in BMI and body weight during pregnancy are extremely sensitive to initial weight, it is not enough to adjust the prepregnancy BMI category alone.

The IOM guidelines were based on findings from observational studies focused on associations of GWG with preterm birth, small, and large size for gestational age at birth, cesarean delivery, postpartum weight retention, and childhood obesity [26]. This study provided a valuable complement to the guidelines. And more research is needed to confirm the effect of optimal weight gain recommended by the guidelines on the prevention of GDM.

### Limitation

To obtain accurate body weight, we took the measured weight during the first antenatal examination to calculate the initial BMI. That is because the difference between prepregnancy weight and early pregnancy weight is not significant and is generally assumed to be less than 2 kg. Moreover, the two are often commended to calculate pre-pregnancy BMI and GWG. Therefore, self-reported pre-pregnancy weight was not performed in this study. Although we require all pregnant women to start antenatal examination as soon as possible after confirming pregnancy, the average gestational age of the initial antenatal visit was 10 weeks. Because of this inevitable shortcoming, we can’t accurately evaluate the weight or BMI at the first trimester before 10 weeks.

## Conclusions

BMI in early pregnancy was a risk factor for GDM, while BMI gain before GDM screening was not associated with the risk of GDM. Therefore, the optimal BMI in early pregnancy is the key to preventing GDM.

## Supplementary Information

**Additional file 1: Additional Fig. 1.** Study flow chart. **Additional Fig. 2.** Correlation between BMI gain and baseline BMI in the GDM group and the non-GDM group. **Additional Table 1.** Compare the prevalence of GDM in different weight gain categories. **Additional Table 2.** Compare baseline BMI in the GDM and the non-GDM groups. **Additional Table 3.** Compare weight gain in the GDM and the non-GDM groups. **Additional Table 4.** Odds ratios (95% confidence intervals) of GDM by the effect of weight gain during pregnancy.

## Data Availability

The datasets used and analyzed during the current study are available from the corresponding author on reasonable request.

## Abbreviations

GDM: Gestational diabetes mellitus
BMI: Body mass index
PCOS: Polycystic ovary syndrome
MNT: Medical nutrition therapy
GWG: Gestational weight gain
IOM: Institute of Medicine
TJWCHIS: Tianjin Women and Children Health Information System
FG: Fasting glucose
GCT: Glucose challenge test
OGTT: Oral glucose tolerance test
DIP: Diabetes in pregnancy
SBP: Systolic blood pressure
DBP: Diastolic blood pressure
MAP: Mean arterial pressure
