# Supplementary material for: Relationship between gestational body mass index change and the risk of gestational diabetes mellitus: a community-based retrospective study of 41,845 pregnant women
Source: BMC Pregnancy Childbirth. 2022 Apr 19;22:336. doi: 10.1186/s12884-022-04672-5 (PMC9020000; doi:10.1186/s12884-022-04672-5)
Supplement: Supplementary file 1 — Additional file 1: Additional Fig. 1. Study flow chart. Additional Fig. 2. Correlation between BMI gain and baseline BMI in the GDM group and the non-GDM group. Additional Table 1. Compare the prevalence of GDM in different weight gain categories. Additional Table 2. Compare baseline BMI in the GDM and the non-GDM groups. Additional Table 3. Compare weight gain in the GDM and the non-GDM groups. Additional Table 4. Odds ratios (95% confidence intervals) of GDM by the effect of weight gain during pregnancy. [file 12884_2022_4672_MOESM1_ESM.docx]

**Additional file**

**Additional Fig. 1** Study flow chart


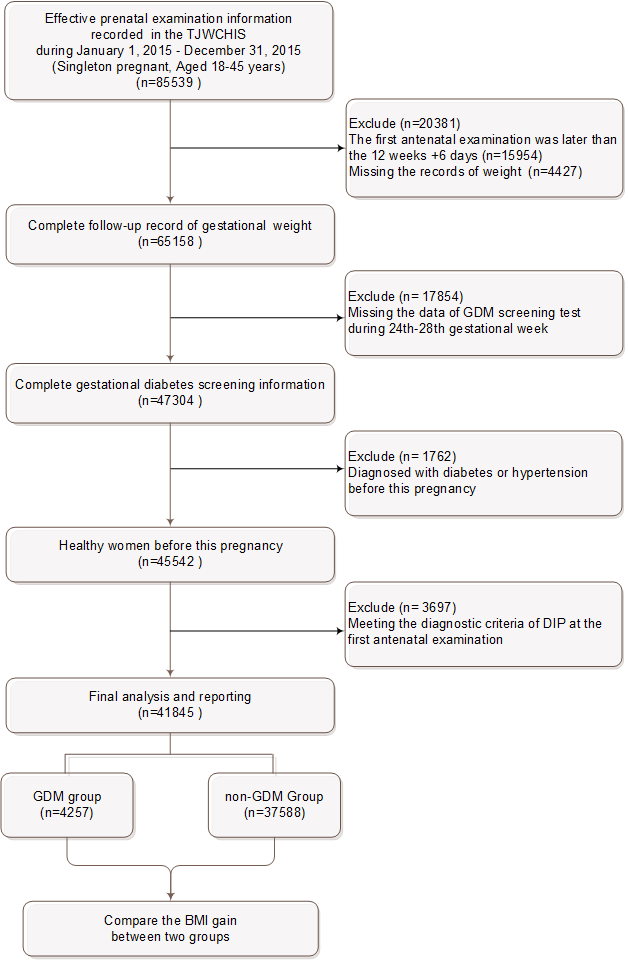


**Additional Fig. 2** Correlation between BMI gain and baseline BMI in the GDM group and the non-GDM group.

Data are mean values. Abbreviation: GDM, gestational diabetes mellitus; BMI, body mass index.

**Additional Table 1.** Compare the prevalence of GDM in different weight gain categories

| Early pregnancy BMI | Weight gain categories | non-GDM group | GDM group | *P*-value |
| --- | --- | --- | --- | --- |
| all BMI | adequate | 8191(91.1%) | 796(8.9%) | <0.001 |
| (n=41845) | insufficient | 6514(89.5%) | 768(10.5%) |  |
|  | excessive | 22883(89.5%) | 2693(10.5%) |  |
| BMI <18.5 kg/m^2^ | adequate | 1034(95.9%) | 44(4.1%) | 0.683 |
| (n=4080) | insufficient | 1025(95.5%) | 48(4.5%) |  |
|  | excessive | 1837(95.2%) | 92(4.8%) |  |
| BMI 18.5-23.9 kg/m^2^ | adequate | 6079(91.6%) | 55.7(8.4%) | 0.026 |
| (n=24963) | insufficient | 3804(91.5%) | 354(8.5%) |  |
|  | excessive | 13105(92.5%) | 1064(7.5%) |  |
| BMI 24.0-27.9 kg/m^2^ | adequate | 736(85.2%) | 128(14.8%) | 0.893 |
| (n=8934) | insufficient | 1033(85.0%) | 182(15.0%) |  |
|  | excessive | 5861(85.5%) | 994(14.5%) |  |
| BMI ≥28.0 kg/m^2^ | adequate | 342(83.6%) | 67(16.4%) | 0.064 |
| (n=3868) | insufficient | 652(78.0%) | 184(22.0%) |  |
|  | excessive | 2080(79.3%) | 794(20.5%) |  |

Data was conducted by *Chi-square* test. Weight gain was evaluated according to the IOM guidelines. It recommended the optimal rate of weight gain at the second trimester was 0.44-0.58, 0.35-0.50, 0.23-0.33, and 0.17-0.27 kg/week in the underweight, normal weight, overweight, and obese groups, respectively. Abbreviation: GDM, gestational diabetes mellitus; BMI, body mass index.

**Additional Table 2.** Compare baseline BMI in the GDM and the non-GDM groups

| Early pregnancy BMI categories | *n* | non-GDM group | GDM group | *t* | *P-value* |
| --- | --- | --- | --- | --- | --- |
| Underweight (<18.5 kg/m^2^) | 4080 | 17.53(0.82) | 17.55(0.83) | -0.32 | 0.747 |
| Normal weight (18.5-23.9 kg/m^2^) | 24963 | 21.20(1.48) | 21.62(1.48) | -12.08 | <0.001 |
| Overweight (24.0-27.9 kg/m^2^) | 8934 | 25.60(1.12) | 25.76(1.12) | -4.59 | <0.001 |
| Obese (≥28.0 kg/m^2^) | 3868 | 30.71(2.45) | 31.14(2.93) | -3.82 | <0.001 |

Abbreviation: GDM, gestational diabetes mellitus; BMI, body mass index.

**Additional Table 3.** Compare weight gain in the GDM and the non-GDM groups

| Early pregnancy BMI | *n* | Weight gain (kg) | | *t* | *P-value* |
| --- | --- | --- | --- | --- | --- |
| (kg/m^2^) |  | non-GDM group | GDM group |  |  |
| <18.50 | 4080 | 7.25 (2.40) | 7.11 (2.53) | 0.810 | 0.418 |
| 18.50 - 19.99 | 6203 | 7.22 (2.53) | 7.12 (2.76) | 0.688 | 0.491 |
| 20.00 - 21.99 | 10253 | 7.14 (2.66) | 7.12 (2.65) | 0.261 | 0.794 |
| 22.00 - 23.99 | 8507 | 7.09 (2.84) | 6.98 (2.85) | 1.115 | 0.265 |
| 24.00 - 25.99 | 5653 | 6.78 (2.95) | 6.78 (2.99) | -0.008 | 0.994 |
| 26.00 - 27.99 | 3281 | 6.18 (3.18) | 6.10 (2.96) | 0.531 | 0.595 |
| 28.00 - 29.99 | 1845 | 5.71 (3.26) | 5.55 (2.86) | 0.911 | 0.362 |
| ≥30.00 | 2023 | 4.81 (3.47) | 4.63 (3.46) | 0.956 | 0.339 |
| *F* |  | 227.04 | 45.24 |  |  |
| *P-value* |  | <0.001 | <0.001 |  |  |

Abbreviation: GDM, gestational diabetes mellitus; BMI, body mass index.

**Additional Table 4.** Odds ratios (95% confidence intervals) of GDM by the effect of weight gain during pregnancy

| Model | Factors | *β* | *OR* | *OR 95% C.I.* | *P*-value |
| --- | --- | --- | --- | --- | --- |
| Univariate analysis | |  |  |  |  |
|  | GWG evaluated by the IOM guideline | | | | |
|  | adequate |  |  |  |  |
|  | insufficient | 0.164 | 1.178 | 1.067-1.302 | 0.001 |
|  | excessive | 0.116 | 1.123 | 1.037-1.217 | 0.005 |
| Multivariate analysis | |  |  |  |  |
| Model 1 | | | | | |
|  | GWG evaluated by the IOM guideline | | | | |
|  | adequate |  |  |  |  |
|  | insufficient | -0.021 | 0.979 | 0.884-1.084 | 0.685 |
|  | excessive | -0.048 | 0.953 | 0.878-1.034 | 0.250 |
| Model 2 | | | | | |
|  | GWG evaluated by the IOM guideline | | | | |
|  | adequate |  |  |  |  |
|  | insufficient | -0.063 | 0.939 | 0.847-1.040 | 0.228 |
|  | excessive | -0.020 | 0.980 | 0.902-1.064 | 0.630 |
| Model 3 | | | | | |
|  | GWG evaluated by the IOM guideline | | | | |
|  | adequate |  |  |  |  |
|  | insufficient | -0.014 | 0.986 | 0.889-1.094 | 0.795 |
|  | excessive | 0.002 | 1.002 | 0.922-1.089 | 0.967 |

GWG evaluated by the IOM guideline according to the WHO BMI categories: underweight (BMI <18.5 kg/m^2^), normal weight (BMI 18.5-24.9 kg/m^2^), overweight (BMI 25.0-29.9 kg/m^2^), and obese (BMI ≥ 30.0 kg/m^2^), respectively.

Model 1: adjusted for early pregnancy BMI;

Model 2: adjusted for early pregnancy BMI, FG in the first trimester, and MAP;

Model 3: adjusted for early pregnancy BMI, FG in the first trimester, MAP, age, multipara, PCOS, history of macrosomia, history of adverse fertility, family history of diabetes, and habitual smoking.

Abbreviation: OR, odds ratio; CI, confidence interval; GDM, gestational diabetes mellitus; BMI, body mass index; GWG, gestational weight gain; FG, fasting glucose; MAP, mean arterial pressure; PCOS, polycystic ovarian syndrome.
